# Quantifying deep neural network uncertainty for atrial fibrillation detection with limited labels

**DOI:** 10.1038/s41598-022-24574-y

**Published:** 2022-11-22

**Authors:** Brian Chen, Golara Javadi, Alexander Hamilton, Stephanie Sibley, Philip Laird, Purang Abolmaesumi, David Maslove, Parvin Mousavi

**Affiliations:** 1 School of Computing, Queen’s University, Kingston, ON Canada; 2 Department of Critical Care Medicine, Queen’s University, Kingston, ON Canada; 3 Department of Electrical and Computer Engineering, University of British Columbia, Vancouver, BC Canada

**Keywords:** Health care, Atrial fibrillation, Cardiology, Information technology, Computer science, Medical research

## Abstract

Atrial fibrillation (AF) is the most common arrhythmia found in the intensive care unit (ICU), and is associated with many adverse outcomes. Effective handling of AF and similar arrhythmias is a vital part of modern critical care, but obtaining knowledge about both disease burden and effective interventions often requires costly clinical trials. A wealth of continuous, high frequency physiological data such as the waveforms derived from electrocardiogram telemetry are promising sources for enriching clinical research. Automated detection using machine learning and in particular deep learning has been explored as a solution for processing these data. However, a lack of labels, increased presence of noise, and inability to assess the quality and trustworthiness of many machine learning model predictions pose challenges to interpretation. In this work, we propose an approach for training deep AF models on limited, noisy data and report uncertainty in their predictions. Using techniques from the fields of weakly supervised learning, we leverage a surrogate model trained on non-ICU data to create imperfect labels for a large ICU telemetry dataset. We combine these weak labels with techniques to estimate model uncertainty without the need for extensive human data annotation. AF detection models trained using this process demonstrated higher classification performance (0.64–0.67 F1 score) and improved calibration (0.05–0.07 expected calibration error).

## Introduction

Atrial fibrillation (AF) is one of the most common cardiac arrhythmias, with a risk of development of 25% for adults over the age of 40^1^. In the intensive care unit (ICU), new onset atrial fibrillation is even more prevalent. New onset AF has been reported in as many as 46% of ICU patients^2^. This includes both patients with a history of AF who develop an episode while in critical care, as well as patients without a prior history that develop AF as a result of other risk factors (e.g. age) and clinical interventions (e.g. application of certain vasopressors)^1^. The etiology of new onset AF in the ICU differs from that of AF as it presents in the general population^3–5^. It is often found in patients with sepsis^6^, but has also been seen with acute respiratory distress syndrome^7^ and acute trauma^2^, as well as after cardiac^8^ and non-cardiac^9^ thoracic surgery. New onset AF is associated with a number of adverse downstream outcomes, including ischemic stroke, heart failure, increased length of ICU stay, and increased mortality risk. In addition to the direct risk of patient harm, delayed recognition and intervention has risks for the quality of healthcare delivery and can potentially lead to much higher costs from interventions that could be prevented with a strategy of early detection and prevention.

The true burden of AF in the ICU has not been thoroughly quantified. New onset AF in particular is difficult to study, as it is difficult to detect in real time. Clinicians at the bedside may be able to identify the arrhythmia when it is sustained, but paroxysmal (temporary or fleeting) episodes may go unnoticed unless the bedside monitor is under constant scrutiny. Automated detection of new onset AF holds promise for both quantifying the burden of the disease and catching fleeting episodes^1,10,11^. From the perspective of precision medicine, which seeks to create more targeted treatments for individuals or certain groups of patients, this becomes especially important when evaluating interventions for clinical trials^12^. The trend of clinical trials in the ICU has been one of pervasive negative results^13–15^, perhaps in large part due to broad heterogeneity in underlying physiological factors among the patient cohorts included in most trials. Going beyond the one-size-fits-all model and allowing judicious targeting of patients with a particular condition would be highly beneficial for more effective usage of trial resources^16^. This kind of “predictive enrichment” contributes not only to our scientific understanding of conditions like new onset AF, but also helps inform best practices around the standard of care and which interventions might be most effective at addressing it^17^.

Thankfully, the same format used to identify and diagnose AF in the ICU also holds potential for detecting and quantifying its incidence. Electrocardiogram (ECG) telemetry is a staple of ICU monitoring, and already used at the bedside by critical care physicians and nurses to flag patients as having AF. On an ECG, the irregular and rapid beating of the atria present in AF manifests as a missing P-wave (representing disorganized atrial depolarization) and irregularly spaced QRS complexes (i.e. a highly variable R-R or peak-to-peak interval). Being able to leverage the ECG waveform directly has also shown potential for new onset AF quantification^1,11,18^. In this vein, recent work on machine learning models with derived features of ECG waveforms such as heart rate variability has obtained state-of-the-art performance for AF classification^19,20^. Others have sought to apply various deep learning techniques to the problem of ECG interpretation^21^, as such models thrive on large datasets.

The training data used in ML-based AF classifiers tends to be noisy, particularly when derived from ICU patients where sensor drop-off and motion artifact are common. Prior work has generally addressed this noise through the use of traditional signal processing techniques^22^ or designing models robust to input noise^23^. However, there exists an additional layer of uncertainty resulting from the ground-truth labels assigned to training data^24^. These can be challenging to acquire due to the clinical expertise required, and the large number of labels needed for modern deep learning models. For example, a clinical note that an ICU patient experienced an episode of AF may not provide onset and offset times to align with ECG waveform data. For retrospective analyses, expert reviewers may disagree on rhythm diagnoses when brief segments of ECG signal are evaluated in isolation and without clinical context.

**Figure 1:**
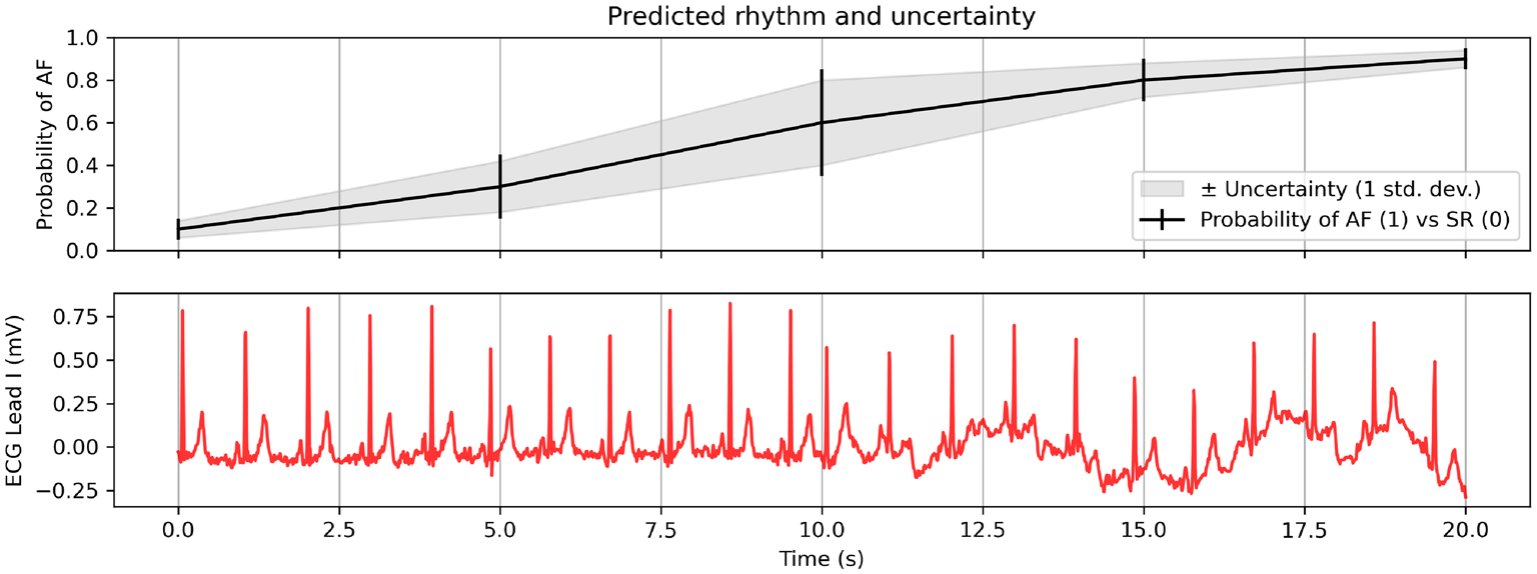
A hypothetical example of how uncertainty estimates can help in interpreting ML model predictions. Here, an AF or sinus rhythm classifier is generating probability estimates for short segments of continuous ECG data. Adding an uncertainty estimate allows us to highlight the transition between different rhythm types, as shown in the raw (unprocessed) signal in red. Depending on the use case, these transition periods could be flagged for further inspection or ignored in favour of less ambiguous time intervals.

Despite achieving state-of-the-art performance in many domains, deep learning models in particular tend to produce unreliable, poorly calibrated confidence estimates for their predictions^25,26^. Whereas a physician might say “I don’t know” when interpreting a noisy record, a deep network would persist in issuing high confidence predictions on possibly ambiguous input data. To address the issue of poor calibration and provide alternative means for quantifying the uncertainty of model predictions, a growing body of literature has investigated uncertainty estimation techniques for deep learning^26–28^. In particular, uncertainty estimation techniques have seen great interest in the healthcare domain for improving confidence estimates, identifying dataset shift and providing richer predictive information beyond simple point estimates of classification performance^29–37^. Quantitative estimates from a ML model may be used to provide richer, more interpretable decision support recommendations to human reviewers (Fig. 1).

While uncertainty estimation techniques may help in mitigating the impact of noise or other artifacts for machine learning models, they do not directly address the issue of acquiring sufficiently large ground truth datasets on which to train said models. By obtaining and training on a larger quantity of “weakly labelled” non-ground truth data, weakly supervised learning techniques can address this dependency^38,39^. Indeed, a combination of weak supervision and uncertain estimation has been applied to generate more accurate, higher quality results for medical image translation^35^ segmentation^32,40^, enable better targeting of ultrasound-guided prostate biopsies^41^, as well as in non-healthcare domains such as improving the data efficiency of image attribute editing for generative models^42^. In this work, we leverage both weak supervision and uncertainty estimation for training and evaluating deep models on streaming ECG telemetry from the ICU. To our knowledge, this is the first work to apply both techniques to waveform data from the ICU, especially in the area of scientific discovery for clinical care. Using weak labels generated from a surrogate model trained on non-ICU data, we demonstrate that training with weak supervision leads to better calibrated AF detection models. We further explore how applying uncertainty estimation improves both performance and calibration of models trained with weak labels. Lastly, we show that training with weak supervision and uncertainty estimation methods allows models to identify both noisy and out-of-distribution (OOD) rhythms.

## Methods

**Figure 2:**
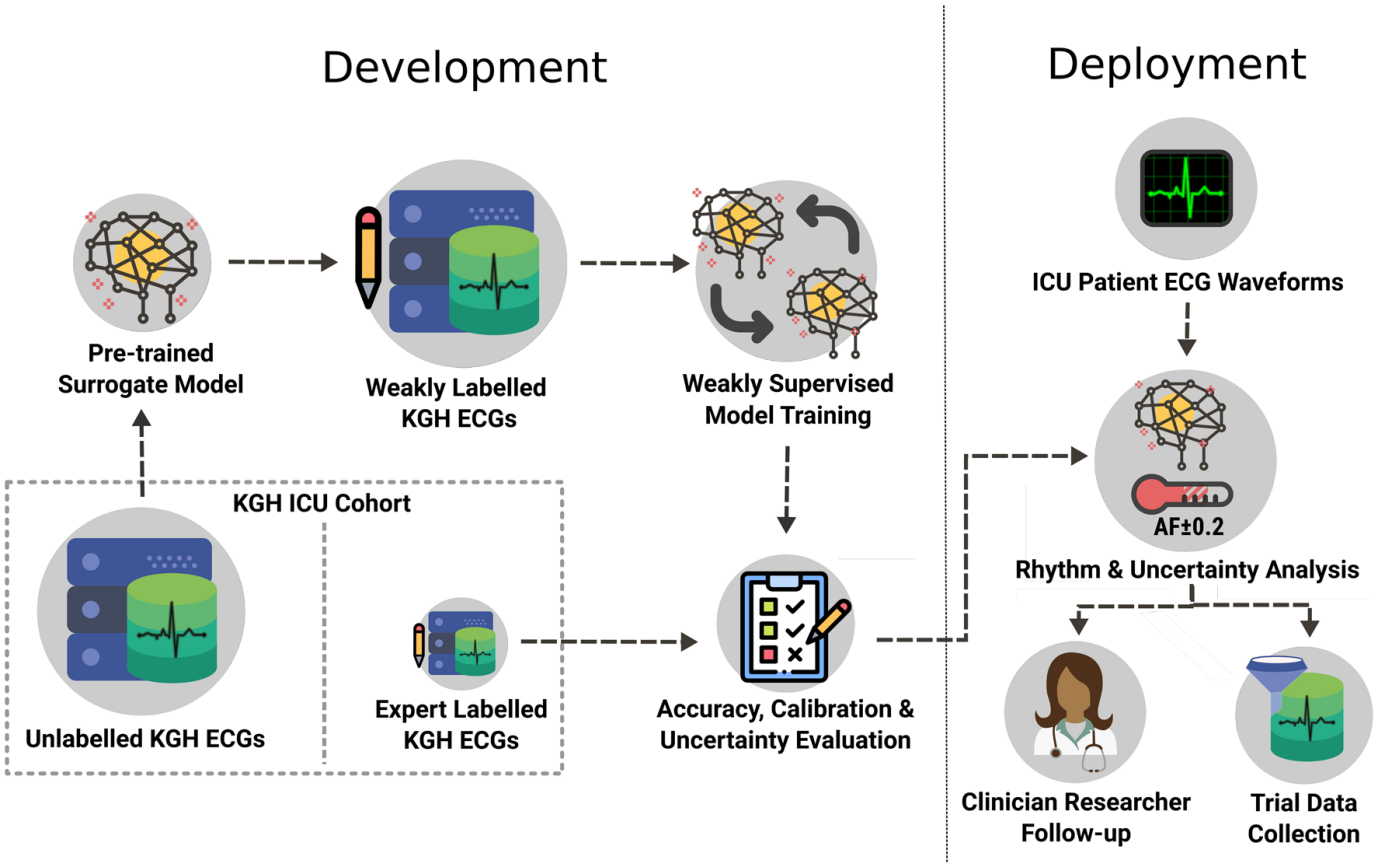
High-level description of the experimental pipeline and desired clinical application. The KGH cohort data were split into a small test set labelled by expert annotators and a much larger, unlabelled training set. Unlabelled ECG waveforms from this cohort dataset were fed into a surrogate model (pre-trained ECG classifier) to generate weak labels for the unlabelled training data. These were then used to train a new model from scratch with weak supervision techniques. Evaluating calibration and model uncertainty has a direct application for deployment (right), as it ensures the model can provide a reliable estimate of its confidence on previously unseen data.

Figure 2 summarizes our experimental procedure. ECG waveforms were extracted from an institutional ICU database. A subset of this data received expert labels, while the remainder were annotated by an arrhythmia classifier pre-trained on external non-ICU data. These model-generated annotations are referred to as “weak labels” because they have not been corroborated by a human expert annotator or some external ground truth data such as a timestamped diagnosis code. Using these weak labels, we trained institution-specific deep AF models with and without weak supervision. Finally, we evaluated the accuracy and reliability of predictions from the trained models, as well as the quality of the uncertainty estimates generated.

### Data extraction and annotation

Our main source of data was collected from bedside monitors in the ICU at Kingston General Hospital (KGH) in Kingston, Ontario, Canada, a 33-bed mixed use medical surgical, neurological, and trauma facility. Over 11TB of physiological waveform and vital sign data were collected from GE Solar monitors using Bedmaster software (Hill-Rom Holdings Inc, Chicago, Ill.) between 2015 and 2020. From this set, we extracted 4 ECG leads (I, II, III and a V1 equivalent) at 240Hz. Patients who were transferred between ICU beds during their stay were excluded in order to ensure admission and discharge times could be aligned reliably, resulting in an anonymized cohort of 1043 patients. The collection and usage of both monitor and bed transfer data was approved by our institutional ethics review board. The collection and use of the data in this study was granted ethical clearance by the Queen’s University Health Sciences & Affiliated Teaching Hospitals Research Ethics Board. This study used archival data that was fully de-identified and collected as part of routine clinical care. The research ethics board therefore waived the need to obtain informed consent. All methods were also developed in accordance with relevant guidelines and regulations.

We extracted two distinct datasets from the KGH cohort. The first comprised 984 patients, from which we randomly sampled a 10-s segment of recorded ECG signal for each patient. The choice of a 10-s segment length is in line with other studies on deep learning based ECG rhythm classification^24,37,43^, and allowed us to reduce the manual burden of annotating each segment by providing an equivalent view to a ECG printout in the ICU. These segments were reserved for human annotation and as a holdout test set for model evaluation. For the second, unlabelled KGH dataset, we extracted an additional 100,722 segments from the entire 1043 patient cohort by sampling 10 s from each hour of each patient’s stay. To reduce possible overlap between segments and increase rhythm diversity, we ensured a minimum of 1-h intervals between each 10-s segment and sampled at most 1000 segments for each patient stay. Segments were rejected if any sensor drop-off was present, but otherwise were not checked for other forms of noise. The mean number of segments per patient was 101, while the median was 57. Because of the variation in per-patient segment counts (e.g. longer stays and poor sensor measurements), we further reduced the maximum segments for each patient based on the upper whisker of a boxplot of the distribution, i.e. 1.5 x the IQR + the upper quartile = 267. This left a total of 84,614 unlabelled segments available for model training.

### Label acquisition and weak label generation

To obtain ground truth labels for the test set segments, we defined a set of 8 disjoint classes for annotation. This included sinus rhythm (regardless of the underlying heart rate), atrial fibrillation (combined with atrial flutter), artificial pacemaker rhythms, and other types of tachy- and bradyarrythmias. An additional class for abstentions was included to track samples where human annotators were unable to clearly identify a specific rhythm type. Annotation was conducted by two critical care physicians experienced in ECG interpretation in an ICU context. 665 of 984 segments were given labels by both annotators, and the overall Cohen’s Kappa inter-rater agreement level was 0.81. A third critical care physician acted as a tiebreaker in cases of disagreements. Because not all 8 classes were identified in significant quantity during annotation, only the three largest (sinus rhythm, atrial fibrillation/flutter and abstentions, amounting to a total of 956 segments) were retained for further analysis in this study while the remaining classes were discarded. In parallel, we also recorded reasons for abstention from the clinician annotators. For all but two segments, the reason given was that the ECG waveform was too unclear or noisy to reliably classify.

To collect additional information on potentially noisy segments, a second round of annotation was conducted with a critical care physician. This involved a simple binary label indicating whether each segment was “clean” or contained noise. To label a segment as noisy, at least 1/4 of the recording had to contain some form of high-frequency artifact or low-frequency baseline wander in the recording. Comments were also collected about specific types of noise present for each noisy sample. Once annotated, the labelled segments were reserved as a holdout set and not used in any subsequent training.

To generate labels for the unlabelled KGH data, we used a surrogate binary classification model trained on the Chapman 12 lead dataset^44^. This dataset of 10,645 patients was sourced from diagnostic ECGs as opposed to ICU telemetry, but was chosen because of the overlap in leads and classes compared to the labelled KGH data. To match the KGH data, we down-sampled the 10 s segments from 500 to 240Hz and only extracted leads I, II, III and V1. The Chapman Sinus Bradycardia, Sinus Rhythm and Sinus Irregularity classes were mapped to our sinus rhythm class, while the Chapman Atrial Flutter and Atrial Fibrillation classes were mapped to our AF class. This left a total of 9508 samples, which were randomly split into training (70%) and validation (30%) components stratified by rhythm class.

Once trained, the label generation model was applied to the unlabelled KGH dataset to predict labels for each sample. These labels were considered “weak” as they did not receive any human verification. Of the 84,614 samples labelled, 55,863 were classified as sinus rhythm and 28,751 as the AF class. Following the same training procedure as the label generation model, we trained a new classifier directly with the weak labels. This model was only able to access the unlabelled KGH data and not the external Chapman dataset.

### Model architecture and training

We made use of the deep CNN architecture described by Goodfellow et al.^45^ for our classification and label generation models. This network comprises 13 blocks of 1D convolutions, batch normalization, ReLU activation and 30% dropout. Dilated max-pooling is selectively applied at blocks 6 and 11, while per-block dilation is gradually increased from 1 to 8. Global average pooling and a final linear layer are used to accommodate variable-length inputs and generate final outputs for classification, respectively. To encode the target variable, we used a binary label of 0 = sinus rhythm and 1 = AF with binary cross-entropy as the loss function. We used a batch size of 128, and the Adam optimizer^46^ with a learning rate of 0.01 and weight decay of 0.0001. Unless otherwise specified, models were trained for a maximum of 200 epochs with early stopping after 10 epochs of no loss improvement. Model creation and training were implemented using PyTorch^47^ and PyTorch Lightning^48^.

**Figure 3:**
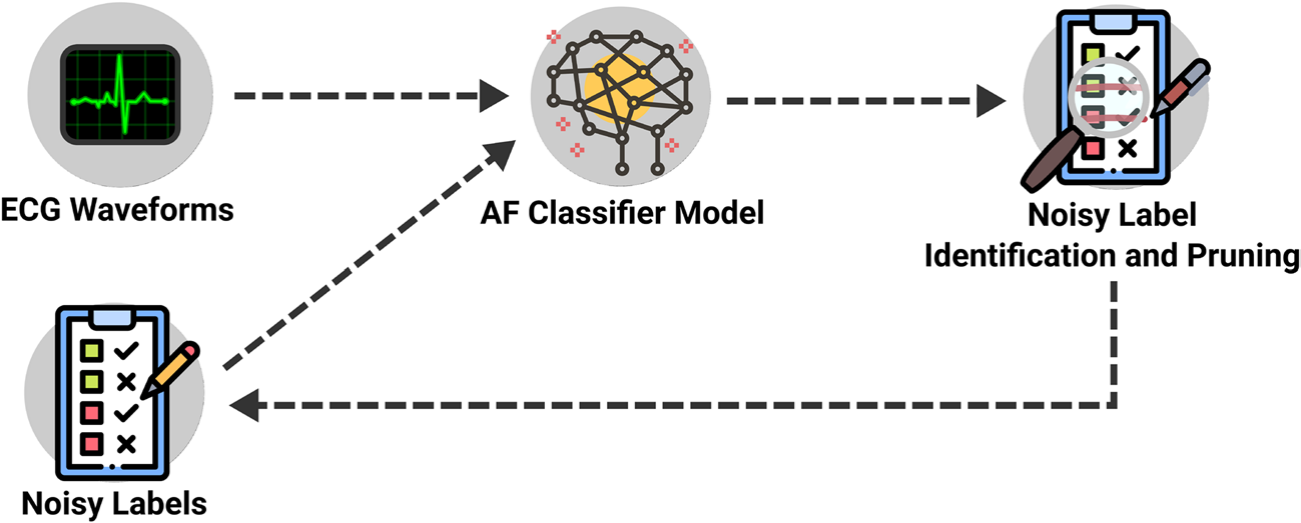
Overview of weak supervision with confident learning. Network predictions are combined with weak labels to generate the confident joint, Which in turn is used to identify potentially incorrect labels for cleaning. The set of cleaned labels is used for further training.

### Label cleaning with confident learning

In addition to training a model directly on the weakly labelled KGH data, we leveraged Confident Learning^49^, a weakly supervised learning technique that mitigates the impact of incorrect labels. Given a set of noisy labels $\widetilde{y}$, Confident Learning estimates the joint distribution between $\widetilde{y}$ and an unknown set of “clean” labels $y^*$ that form the actual ground truth. This joint distribution is approximated by aggregating normalized counts of potentially misclassified labels for each class into a (in this case, 2x2) square matrix called the confident joint, $C_{\widetilde{y},y^*}$. Much like a confusion matrix, the diagonal of the confident joint represents correct label assignments. To construct $C_{\widetilde{y},y^*}$, each sample is compared against the mean predicted probability of its class, $t_j$,

1 $$\begin{aligned} t_j = \frac{1}{N_j} \sum ^{N_j}_{i=1} p(\widetilde{y}_i=j|x_i,\theta ) \end{aligned}$$

where $N_j$ is the number of samples for class *j* and $p(\widetilde{y}_i=j|x_i,\theta )$ is the predicted probability of *j* for a given sample.

Once constructed, the confident joint can be used to find labels that do not meet per-class thresholds and thus are likely candidates for being misclassified. These candidate samples labels are then pruned from the dataset and are no longer present in subsequent training epochs (Fig. 3). Using the cleanlab library^49^, we applied this iterative label cleaning every 3 epochs until the end of training. For a more detailed analysis on the choice of cleaning interval, see Chen et al.^50^.

### Uncertainty estimation

One framework for interpreting uncertainty is that of aleatoric vs epistemic uncertainty^51^. Aleatoric uncertainty arises from the intrinsic stochasticity that manifests when measuring a real world process or phenomenon. As long as the underlying distribution or behaviour of the system under observation does not shift, this form of uncertainty is generally considered irreducible: that is, collecting more data will not allow one to reduce the aleatoric uncertainty. One important subset of aleatoric uncertainty for machine learning applications is heteroscedastic uncertainty, which occurs when different inputs result in different levels of uncertainty in outputs. For example, noisier input data will tend to result in more uncertain model predictions^27^. Epistemic uncertainty, on the other hand, arises from a lack of knowledge of the true distribution of the outcome. Because collecting more data and incorporating more knowledge into the modelling process can mitigate epistemic uncertainty, it is also often referred to as model uncertainty. When developing machine learning models, epistemic uncertainty can be used to find and understand OOD samples that deviate from the training data^27^.

Uncertainty estimation techniques seek to identify and quantify both aleatoric and epistemic uncertainty in the pursuit of enhancing predictive and decision-making power. We leveraged techniques for estimating both aleatoric and epistemic uncertainty in our AF models. Although there are a wide range of existing uncertainty estimation methods, we focused on those which would not require invasive changes to the existing classification models (e.g. converting into a Bayesian neural network and using variational inference) or high compute overhead (e.g. a full ensemble model). To that end, we selected Monte Carlo Dropout^52^ and test-time augmentation to generate epistemic and aleatoric uncertainty estimates respectively. Both types of uncertainty were tested separately and in combination.

**Figure 4:**
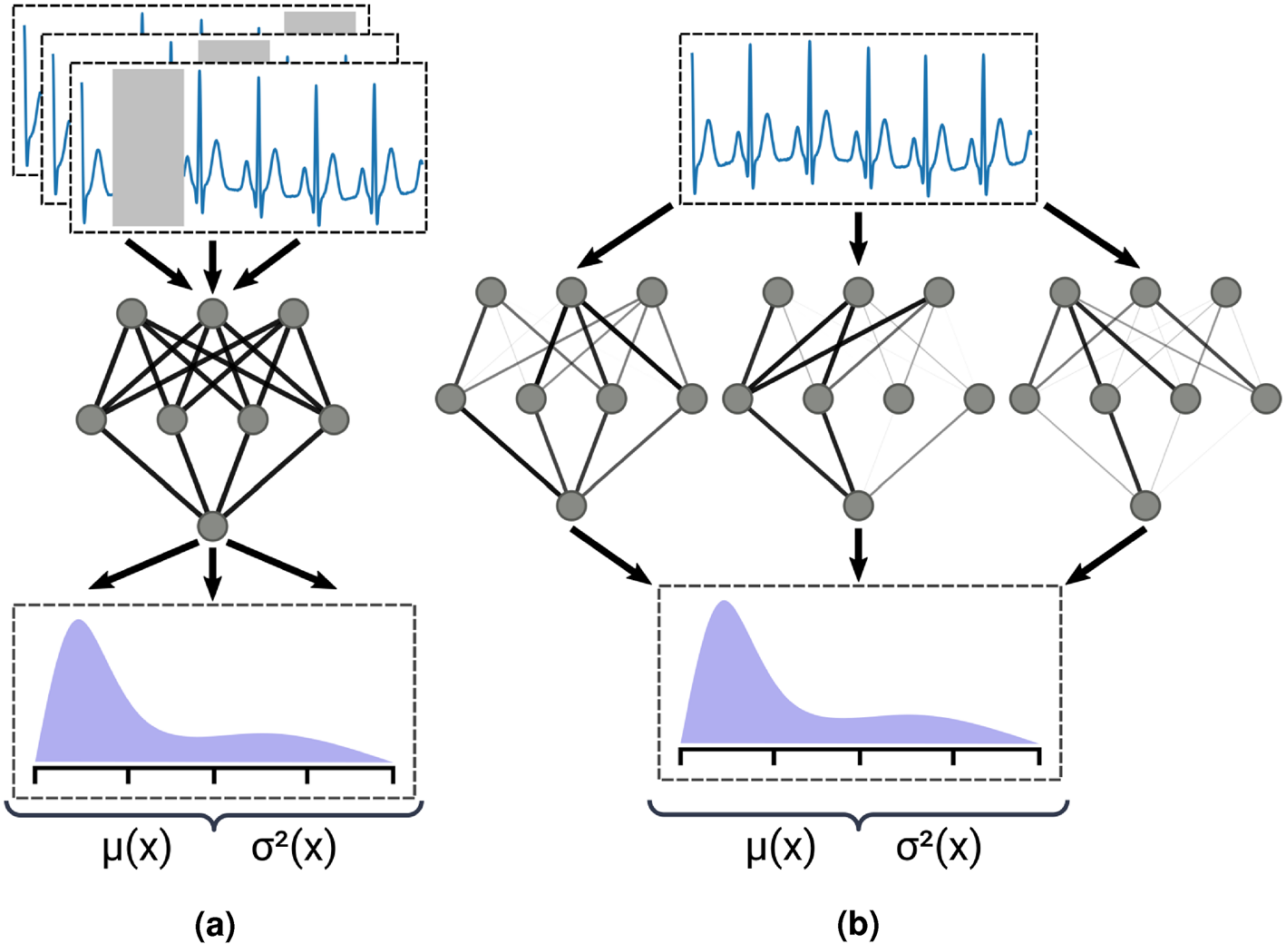
Visual comparison of aleatoric and epistemic uncertainty estimation techniques. (**a**) Test-time augmentation randomly samples multiple views of each input. (**b**) Monte Carlo dropout samples multiple configurations of the model weights by randomly excluding a subset of neurons.

#### Test-time augmentation

We employed test-time augmentation to capture aleatoric uncertainty present in our data. While data transformations and augmentations are frequently used when training deep neural networks but disabled during testing, test-time augmentation enables them during evaluation as well. By creating differently augmented views of each input and feeding them through a model, this approach can collect a set of outputs to combine into a more robust aggregate (or pseudo-ensemble) prediction on a single input. This aggregate prediction is derived by averaging the set of predicted probabilities. The multiple collected predictions can also be used to create a distribution of predicted probabilities, from which we can derive a sample standard deviation to use as an uncertainty estimate. If the model outputs a wide range of probabilities for slightly augmented views of the same inputs, then this estimate will reflect a higher level of uncertainty. For our augmentations on ECG data, we applied a 10% random masking on the input signal (Fig. 4a). This approach has been shown to be effective for aleatoric uncertainty estimation in medical imaging^40^, as well as for other augmentation-reliant tasks such as self-supervised learning^53^.

#### Monte Carlo dropout

In order to quantify the level of epistemic uncertainty in our models, we used Monte Carlo dropout (MCDropout)^52^. While dropout layers in a network are usually disabled during inference, MCDropout keeps them active during inference and accumulates outputs from multiple iterations (Fig. 4b). This is equivalent to performing variational inference via Monte Carlo integration over the distribution of weights in the network and the predictive posterior distribution of the predicted outputs. As with test-time augmentation, we can derive an average prediction and epistemic uncertainty estimate from this distribution.

### Evaluating performance and calibration

Performance evaluations were conducted on three different model configurations:

- Label generation model: the pre-trained surrogate model used to generate weak labels on the KGH dataset.
- Weak labels only: a model trained on the weakly labelled KGH data. This training was fully supervised and did not include any weak supervision.
- Confident learning: a model trained on the weakly labelled KGH data, but with the weak supervision via confident learning.

Additionally, we compared against a reproduced version of the state-of-the-art two step algorithm described by Bashar et al.^20^. Because this algorithm uses a set of binary decision rules, it is unable to provide a confidence score or uncertainty estimate. As such, we include it only as a baseline against which to compare AF classification performance. AF identification performance was evaluated using standard classification metrics (F1 score, precision/PPV, recall/sensitivity, and specificity). Because there is a class imbalance between the positive (AF) and negative (sinus rhythm) classes, these metrics ensure that models are not simply predicting the majority class for all samples by assigning equal weight to minority class and penalizing any AF samples which are misclassified.

We made use of three metrics to quantify calibration and the quality of model predictions. Negative Log Likelihood (NLL) is commonly used as part of a loss function to train/optimize on, but is also a proper scoring rule^54^ that can assess the quality of model predictive uncertainty. The Brier score^55^ is another proper scoring rule that measures the accuracy of predicted probabilities. formulated similarly to the mean squared error between predicted probabilities $p(y|x,\theta )$ and binary (i.e. discrete) labels *y*.

2 $$\begin{aligned} Brier\,score = \frac{1}{N}\sum ^{N}_{i=1}(p(y_i|x_i,\theta ) - y_i)^2 \end{aligned}$$

Instead of using a continuous function, expected calibration error (ECE) subdivides the range of predicted probabilities into equally-sized bins and compares the positive class accuracy to the overall predicted confidence in each bin.

3 $$\begin{aligned} ECE = \sum _{b \in B} \frac{|b |}{N} |acc(b) - conf(b)| \end{aligned}$$

Where *B* is the set of bins, *N* is the total number of samples, *acc*(*b*) is the prevalence of the positive class $y=1$ in bin *b*, and *conf*(*b*) is the mean predicted probability $\frac{1}{|b|}\sum ^{|b|}_{i=1} p(y_i=1|x_i,\theta )$. For a discussion on the relative merits and trade-offs of each metric, see Ovadia et al.^26^.

In addition to classification performance and calibration, we also compared uncertainty estimates between models and on different subsets of the ground truth KGH data. One focus was on whether models could effectively differentiate between segments labelled as clean or noisy. To evaluate human-model alignment on abstentions, we compared uncertainty levels for the sinus rhythm and AF classes (considered in-distribution) against samples on which human annotators abstained (considered OOD because the weakly-labelled training dataset did not have any “abstain” labels). Statistical comparisons were conducted with a two-tailed Mann-Whitney U test^56^ to account for non-normality. Bonferroni-Holm correction with a p-value of 0.01 was used to account for comparisons made during multiple hypothesis testing.

## Results

**Figure 5:**
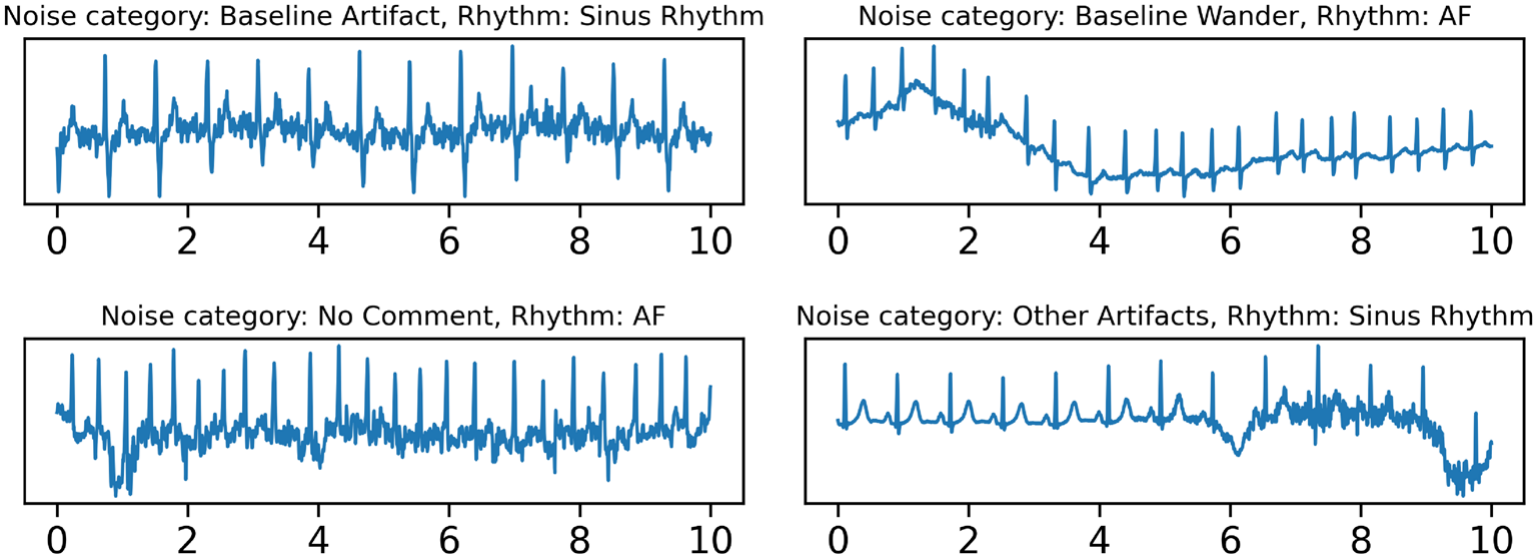
Example, unprocessed segments demonstrating different types of noise in the labelled KGH dataset. “No comment” indicates that while a record was identified to be noisy, there was no specific definition or terminology that clearly applied to the type of noise present. Only lead I is shown.

Of the 984 labelled KGH ECG segments 746 (76%) were identified as sinus rhythm and 123 (13%) as the AF class. Correspondingly, 123 patients had at least one segment labelled as the AF class. Abstentions by clinician annotators were the next largest class at 87 (9%). Of these abstentions, 85 were attributed to the source signal having too much noise (excluding common sources such as baseline wander) to interpret. Similarly, clinician annotators identified 263 (27%) of segments as having some noise by our criteria, with the largest concentration in the abstain class as expected. Figure 5 contains representative examples from this noisy subset. A full summary of the number of segments for each rhythm class and per-class counts of noisy segments may be found in Supplementary Table S1.

Table 1 summarizes the sinus rhythm versus AF class binary classification performance and calibration of the baseline and all three deep learning models tested on the labelled KGH dataset. While the baseline was most effective at finding false positives (PPV 0.73, specificity 0.92), it demonstrated a far lower sensitivity (0.48) and thus lower overall classification performance (F1 score 0.58). In contrast, almost all tested configurations of the three deep learning models were consistently more sensitive, but less precise in predicting the AF class. The two low-sensitivity outlier configurations (training directly on weak labels without any uncertainty estimation or only aleatoric uncertainty estimation) also had lower F1 scores than the baseline (0.53 and 0.54 respectively).

**Figure 6:**
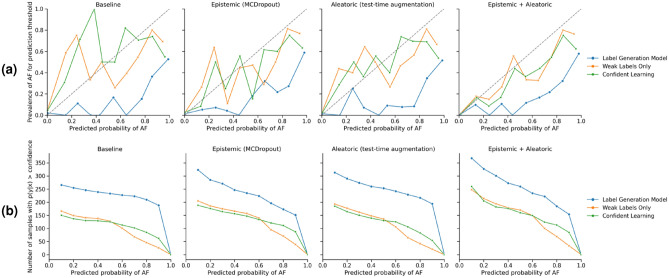
Calibration curves for all three models with various uncertainty estimation techniques applied, as compared to their fully certain baselines. (**a**) reliability curve of predicted probability (confidence) thresholds and the prevalence of the positive AF class from 0 (100% probability of sinus rhythm) to 1 (100% probability of the AF class). The dashed gray line represents a perfect calibration curve. (**b**) confidence versus number of samples with probability greater than the confidence threshold.

Figure 6 shows calibration plots and reliability diagrams for each of the three types of models: the surrogate label generation model trained on external data and used to create weak labels for the unlabelled KGH dataset, the model trained directly on the weak labels in a fully supervised fashion, and the model trained with weak supervision (confident learning) on the weak labels when using different types of uncertainty estimation. The types of uncertainty estimation are “always confident”, which is a fully confident prediction from the network (i.e. a simple point estimate without any uncertainty estimate), epistemic uncertainty (MCDropout), aleatoric uncertainty (test-time augmentation), and a combination of both uncertainty estimation techniques. The reliability plot of calibration curves measures the reliability of a classifier’s estimates. For binary classifiers, This is done by comparing them to the actual prevalence of the positive class being predicted. A perfectly calibrated model will have perfect alignment between predicted and true probabilities. That is, low confidence predictions should correspond to low true likelihoods and vice versa. Perfect calibration is represented by the gray dashed line. The label generation model exhibits the worst calibration overall, with an ECE of 0.14-0.16. This corresponds to an F1 score range of 0.58-0.62. On the reliability curve, this model is the most overconfident about predicting the AF class, as demonstrated by falling under the perfect alignment line. This is reflected in a higher false positive rate, as quantified by lower precision scores (0.43-0.47). The model trained directly on weak labels had the best calibration at 0.05-0.09 ECE. The high end of the F1 score was higher than the label generation model at 0.64, but the low end was lower at 0.53. This was mostly due to a lower sensitivity. Notably, MCDropout on its own and with test-time augmentation improved both classification scores (F1, precision and recall) and calibration. Adding MCDropout also led to consistently higher sensitivity compared to no uncertainty estimation or only using test-time augmentation. The model using confident learning showed similar calibration scores to the one without weak supervision (0.06-0.08 ECE), but with more consistent performance and the highest classification performance overall (0.63-0.67 F1). For both models, uncertainty estimation improved calibration over the baseline, as shown by better alignment on the reliability plot.

The confidence vs number of samples diagram (Fig. 6) shows a similar view to the reliability plot. Here, we again see the binned confidence thresholds, but compared against the number of true (in this case AF class) samples that were actually predicted by the model with a confidence higher than each threshold. As with the reliability plot, this number should decrease at a more consistent rate as the confidence threshold increases if the model is well-calibrated. Here too, we see that introducing uncertainty estimation can have an impact on the alignment of confidence (predicted probabilities) and true likelihood. There is also a sharper drop-off at the highest confidence level, which reflects a bias towards very confident predictions. The other models show a similar slope down to this highest bin, but due to starting with a lower confidence baseline, do not have the same bias towards high confidence predictions.

**Table 1:** Classification and calibration metrics for the baseline and all three deep learning models on labelled sinus rhythm and AF class samples from the KGH dataset.

Model	Uncertainty method	F1	PPV	Sensitivity	Specificity	ECE	Brier	NLL
Bashar et al.^20^	None	0.58	**0.73**	0.48	**0.92**	–	–	–
Label generation model	Always confident	0.62	0.47	0.89	0.84	0.15	0.14	0.73
	Epistemic	0.62	0.47	0.90	0.84	0.14	0.13	0.49
	Aleatoric	0.59	0.44	0.90	0.82	0.16	0.15	0.71
	Epistemic + aleatoric	0.58	0.43	**0.91**	0.82	0.16	0.14	0.52
Weak labels only	Always confident	0.53	0.52	0.54	0.86	0.09	0.10	0.40
	Epistemic	0.64	0.57	0.72	0.88	0.06	**0.08**	0.28
	Aleatoric	0.55	0.52	0.58	0.87	0.07	0.09	0.30
	Epistemic + aleatoric	0.64	0.55	0.76	0.88	**0.05**	**0.08**	**0.25**
Confident learning	Always confident	0.63	0.62	0.63	0.89	0.08	0.09	0.42
	Epistemic	0.66	0.61	0.72	0.89	0.07	**0.08**	0.33
	Aleatoric	0.64	0.62	0.66	0.90	0.06	0.09	0.32
	Epistemic + aleatoric	**0.67**	0.59	0.77	0.89	0.06	**0.08**	0.30

*PPV* positive predictive value or precision; *ECE* expected calibration error; *NLL* negative log-likelihood.

The best scoring model for each metric is shown in bold.

**Figure 7:**
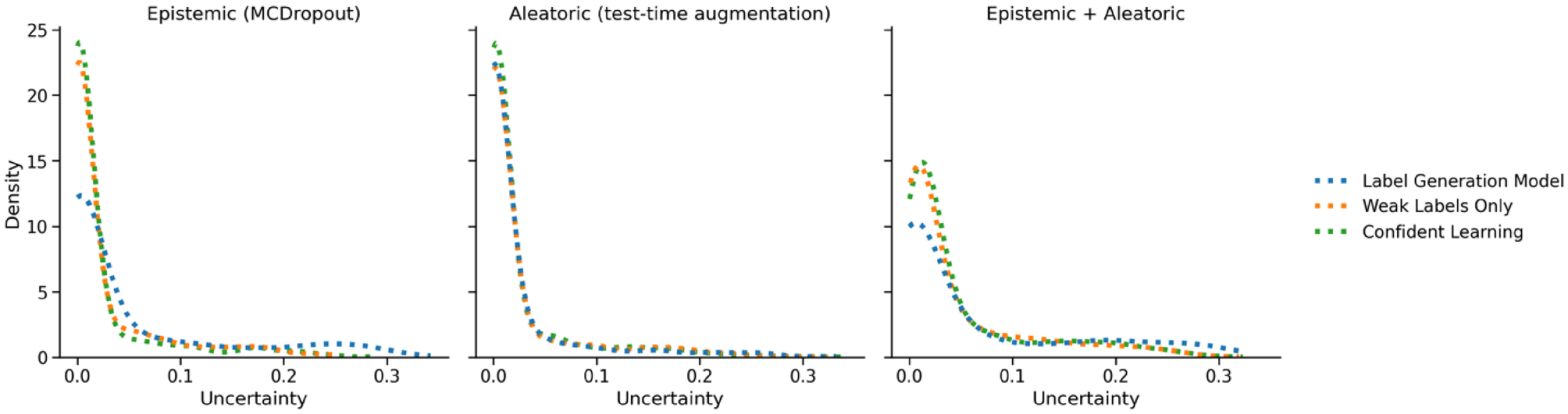
Density plots of uncertainty estimates using both MCDropout and test-time augmentation.

Using MCDropout resulted in better calibration than test-time augmentation for noisy samples, while also maintaining better performance. However, both uncertainty estimation techniques were able to improve upon the fully confident baseline. Just as with the overall test dataset, models trained with weak labels were better calibrated (0.16-0.19 ECE with confident learning, 0.16-0.20 without) than the label calibration model (0.24-0.28 ECE). There was a significant difference between the distribution of uncertainty estimates for noisy samples and clean samples for all three models ($p < 2\times 10^{-9}$). Both aleatoric, epistemic and a combination of both uncertainties were effective at generating these distributions. As Fig. 7 shows, models trained with weak labels generated distributions skewed towards higher uncertainty when provided with noisy samples.

**Figure 8:**
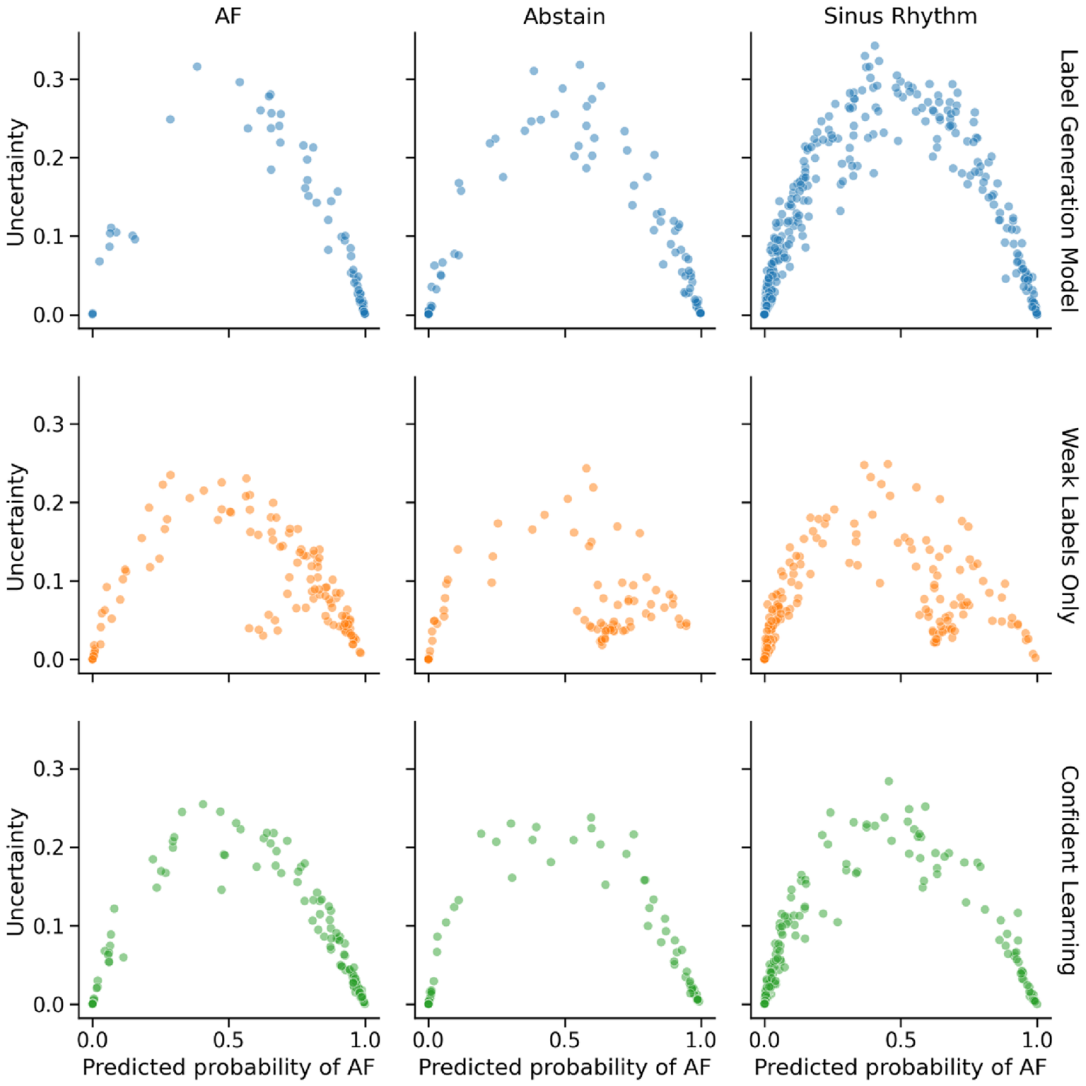
Comparison of model uncertainty versus confidence on in-distribution classes (AF, sinus rhythm) and out of distribution class (Abstain) when using both aleatoric and epistemic uncertainty estimation. All models show an association between high uncertainty and low confidence (i.e. neither definitively the AF nor the sinus rhythm classes). Compared to training with weak labels directly, Confident Learning avoids assigning low uncertainties to more non-confident samples that generally lean towards the AF class.

Because out of distribution samples do not fit within the existing binary class list, we rely on comparing the distribution of uncertainty estimates between in and out of distribution classes. Figure 8 compares predicted probabilities (i.e. confidence) versus uncertainty estimates for the three classes under test. With the expectation that higher uncertainty should be correlated with lower confidence, we see that models generally report higher uncertainty when they are unable to output a clear prediction between sinus rhythm and the AF class. As seen with the relative precision and recall scores, the label generation model is more prone to mistaking sinus rhythm as the AF class, while the models trained with weak labels may do the opposite. All models reported a significant difference between in-distribution and OOD data ($p < 0.0005$) when using test-time augmentation. However, models were only able to differentiate between sinus rhythm and abstentions when MCDropout or a combination of techniques were used.

## Discussion

In this study, we investigated the feasibility of using uncertainty estimation for rhythm classification on ECG data from ICU telemetry. In the context of scientific discovery for both retrospective studies and clinical trials in critical care, uncertainty estimation with weak supervision may be used as a tool for large scale data mining of arrhythmias like atrial fibrillation. The tools presented here could be used as aids for informing clinical decisions, for example by ensuring models are properly calibrated without the need for a significant quantity of testing data, or by using uncertainty to specify the tolerance for false positives or missed samples when identifying AF. Specifically, our models provided higher sensitivity than previous algorithms, albeit at the expense of somewhat lower specificity. Such performance characteristics might well be advantageous in real world applications such as screening for enrolment in clinical trials, where one wants to identify as many research participants as possible, even if some additional analysis is required to confidently identify true AF.

Uniquely, we did not use any human-labelled training data from our institutional dataset as training data. Instead, our classifiers relied on weak labels generated from a pre-trained surrogate model. Prior work has noted the importance of analyzing epistemic uncertainty in particular when limited data are available, as even highly parameterized models such as deep neural networks have fewer samples with which to estimate the true distribution of the data^37,51^. In a similar vein, we found models trained with only weak labels exhibited more desirable performance and uncertainty estimates in almost all comparisons.

Our findings suggest that uncertainty estimation with weak supervision can help improve calibration at similar levels of performance. The label generation model predicts the AF class with higher confidence than both models trained with weak labels. Instead, models trained with weak labels are more likely to make low confidence predictions for the AF class (i.e. high confidence predictions with sinus rhythm). This matches the nature of the KGH dataset and ICU population, where sinus rhythm is by far the dominant rhythm type and the AF class is less than 15% of segments observed in the test data. As with the reliability curve, these biases in model predictions are reflected in the trade-offs made for precision and recall. Thus, using weak labels helps with calibration, and leads to more reliable predictions.

Using uncertainty estimation also allows for better calibration on noisy data compared to a simple, fully confident point estimate from the same model. Likewise, models are better able to distinguish between in distribution data and OOD data, namely sinus rhythm/AF versus expert annotator abstentions. This is expected for the label generation model, as it could not have encountered any data that was too difficult for humans to annotate during training due to our experimental design. Conversely, the presence of samples so noisy as to be non-interpretable by expert clinicians in our labelled KGH data indicates that similar data is likely present in the weakly-labelled training data as well. As such, training with these weak labels may result in OOD data appearing more in-distribution for the model. However, our findings suggest that the label cleaning procedure in confident learning can mitigate this. Compared to training with the weak labels directly, models using confident learning did not output a significant number of low confidence yet low uncertainty predictions.

Comparing techniques that use weak labels, using weak supervision with confident learning achieves superior performance to training without confident learning. Confident learning appears to have the best balance of good performance, calibration and useful uncertainty estimates. The label cleaning helps to reduce the amount of disruption caused by weak labels on all of these factors, while the larger, more relevant training set allows for more reliable predictions on KGH telemetry data. Furthermore, using weak supervision via confident learning further improved upon training with weak labels directly. This was exemplified by the relative uncertainty of both models on KGH AF class data compared to sinus rhythm, which matched the higher proportion of noise in the AF class. In terms of the efficacy of the uncertainty estimation methods themselves, there was no universally superior method between epistemic uncertainty estimation with MCDropout and aleatoric uncertainty estimation with test-time augmentation. Combining both types of uncertainty is also beneficial in certain cases (including for improved performance and calibration when using weak labels), but not for every case. As our dataset contains a similar number of samples as the larger ones used by Vranken et al.^37^, we second their suggestion that more experimentation is required to understand how the relative importance of modelling epistemic and aleatoric uncertainty changes with even larger ECG datasets.

As with any machine learning project in a high-risk domain such as healthcare, creating well-performing models is only the first step on the road to proper clinical deployment. Breadth-wise, we focused on one condition (AF) in a single clinical setting (the ICU) and using one data modality (ECG). The relatively small size of our known patient cohort means that evaluation has been necessarily limited to a slice of the local patient population at KGH. For example, despite not trying to directly classify rhythms other than sinus rhythm and AF, having additional examples of minority classes such as pacemaker rhythms or even bigemeny/trigemeny would have allowed us to conduct a thorough analysis of out-of-distribution uncertainty. Notably, we were also not able to stratify results by certain demographics such as age or gender to ensure that the models were not disadvantaging underrepresented sub-populations^57^. This and additional clinical data integration would be required to create a complete picture of model performance. Our approaches have only been tested on data from one institution and may be influenced by specific attributes of our regional patient population as well as local practice patterns. On the algorithmic front, the question of what machine learning techniques (and specifically which deep learning models) work best for AF and other rhythm detection from ECG remains an open one^24^ that we did not explore at length. Even on the more specialized areas of learning with limited data and uncertainty estimation, we explored only a subset of the possible techniques that have been successfully applied to other healthcare and non-healthcare related machine learning problems^29^.

Future work will focus on development evaluation of these methods with closer to real time data, whether that be more recent retrospective data from the ICU or in silent mode evaluation (not directly making clinical decisions) for live monitoring ECG telemetry. Another avenue we wish to explore is the integration of other sources of weak labels. Although we utilized the same model architecture for both label generation and the final AF classifier, the former could use alternative state-of-the-art approaches which rely on labelled training data^20,43,58^. Alternative representations of the ECG signal such as time-frequency spectrograms have also been shown to be effective for arrhythmia detection models^59^. Beyond models pre-trained on external, non-telemetry datasets, it may be possible to leverage associated clinical data such as lab tests and recorded applications of antiarrhythmic drugs. While these are generally not of a high enough time precision to work as direct labels, they may well have value as weak labels on a surrounding window of data, which can then be narrowed down and cleaned with weak supervision techniques and uncertainty estimation. On the modelling side, more complex uncertainty techniques such as using snapshot ensembles^60^ may help further improve model calibration and uncertainty estimate quality.

## Supplementary Information

Supplementary Table 1.

## Data Availability

The KGH ECG dataset analyzed during the current study is not publicly available due to restrictions on patient privacy and confidentiality, but may be available in de-identified form from the corresponding author on reasonable request and with permission of the institutional review board. The third-party Chapman ECG dataset is available on figshare at https://figshare.com/collections/ChapmanECG/4560497/1.
