# Supplementary material for: Quantifying deep neural network uncertainty for atrial fibrillation detection with limited labels
Source: Sci Rep. 2022 Nov 22;12:20140. doi: 10.1038/s41598-022-24574-y (PMC9684456; doi:10.1038/s41598-022-24574-y)
Supplement: Supplementary file 1 — Supplementary Table 1. [file 41598_2022_24574_MOESM1_ESM.docx]

| **Rhythm Class** | **Count** | **Noisy Samples** |
| --- | --- | --- |
| Sinus Rhythm | 746 | 127 |
| Atrial Fibrillation/Atrial Flutter | 123 | 44 |
| Pacemaker | 20 | 5 |
| Bigemeny and Trigemeny | 3 | 1 |
| Ventricular Tachycardia/Fibrillation | 0 | 0 |
| Other Tachyarrythmia | 0 | 0 |
| Other Bradyarrythmia | 5 | 1 |
| Non-diagnostic (Abstentions) | 87 | 85 |
| **Total** | 984 | 263 |

**Supplementary Table S1.** Counts of segments across all ECG rhythm classes in the annotated KGH data and the number of samples identified as noisy for each class.
